# Uremic Pruritus, Dialysis Adequacy, and Metabolic Profiles in Hemodialysis Patients: A Prospective 5-Year Cohort Study

**DOI:** 10.1371/journal.pone.0071404

**Published:** 2013-08-06

**Authors:** Mei-Ju Ko, Hon-Yen Wu, Hung-Yuan Chen, Yen-Ling Chiu, Shih-Ping Hsu, Mei-Fen Pai, Ju-YehYang, Chun-Fu Lai, Hui-Min Lu, Shu-Chen Huang, Shao-Yu Yang, Su-Yin Wen, Hsien-Ching Chiu, Fu-Chang Hu, Yu-Sen Peng, Shiou-Hwa Jee

**Affiliations:** 1 Department of Dermatology, Ren-Ai Branch, Taipei City Hospital, Taipei, Taiwan; 2 Department of Internal Medicine, Far Eastern Memorial Hospital, New Taipei City, Taiwan; 3 Department of Nursing, Far Eastern Memorial Hospital, New Taipei City, Taiwan; 4 Department of Dermatology, National Taiwan University Hospital and National Taiwan University College of Medicine, Taipei, Taiwan; 5 Department of Internal Medicine, National Taiwan University Hospital and National Taiwan University College of Medicine, Taipei, Taiwan; 6 Graduate Institute of Clinical Medicine, National Taiwan University, Taipei, Taiwan; 7 Graduate Institute of Epidemiology and Preventive Medicine, National Taiwan University, Taipei, Taiwan; 8 School of Nursing, National Taiwan University, Taipei, Taiwan; Universidade de São Paulo, Brazil

## Abstract

**Background:**

Uremic pruritus is a common and intractable symptom in patients on chronic hemodialysis, but factors associated with the severity of pruritus remain unclear. This study aimed to explore the associations of metabolic factors and dialysis adequacy with the aggravation of pruritus.

**Methods:**

We conducted a 5-year prospective cohort study on patients with maintenance hemodialysis. A visual analogue scale (VAS) was used to assess the intensity of pruritus. Patient demographic and clinical characteristics, laboratory parameters, dialysis adequacy (assessed by Kt/V), and pruritus intensity were recorded at baseline and follow-up. Change score analysis of the difference score of VAS between baseline and follow-up was performed using multiple linear regression models. The optimal threshold of Kt/V, which is associated with the aggravation of uremic pruritus, was determined by generalized additive models and receiver operating characteristic analysis.

**Results:**

A total of 111 patients completed the study. Linear regression analysis showed that lower Kt/V and use of low-flux dialyzer were significantly associated with the aggravation of pruritus after adjusting for the baseline pruritus intensity and a variety of confounding factors. The optimal threshold value of Kt/V for pruritus was 1.5 suggested by both generalized additive models and receiver operating characteristic analysis.

**Conclusions:**

Hemodialysis with the target of Kt/V ≥1.5 and use of high-flux dialyzer may reduce the intensity of pruritus in patients on chronic hemodialysis. Further clinical trials are required to determine the optimal dialysis dose and regimen for uremic pruritus.

## Introduction

Uremic pruritus is a common and intractable symptom in patients on chronic hemodialysis [1], [2]. It causes serious discomfort and skin damage, negatively affects the quality of life, and may be associated with sleep disturbance, inflammation, and higher mortality [3], [4]. The pathophysiology of uremic pruritus is complex. Previous studies have shown that xerosis, divalent ions, calcium-phosphate product, C-reactive protein, hepatitis, hyperparathyroidism, immune derangement, and opioid system alternation may be associated with uremic pruritus [5]–[13]. However, there has not been a consensus reached among those studies, and uremic pruritus remains poorly characterized. Because of insufficient understanding for uremic pruritus, current therapeutic options for uremic pruritus are limited and unsatisfactory [14], [15].

Many patients have a prolonged course of uremic pruritus. In particular, 30–60% of patients on maintenance hemodialysis suffer from this problem for longer than one year [1], [16]. And while Mathur described the key features of uremic pruritus and its effect on health-related quality of life in a longitudinal follow-up study [1], the relationships between the intensity of uremic pruritus and biochemical parameters were not investigated. In fact, the intensity of uremic pruritus may vary significantly throughout the course of chronic kidney disease, but factors associated with the severity of pruritus remain unclear due to the lack of relevant longitudinal follow-up studies.

To address this issue, we conducted a prospective 5-year cohort study on hemodialysis patients to identify predictors for the aggravation of pruritus intensity. The relationship of pruritus intensity with metabolic profiles as well as dialysis adequacy was assessed by using change score analysis to consider the individual change in covariates during a longitudinal follow-up.

## Materials and Methods

### Study Participants

A prospective cohort study of patients with maintenance hemodialysis in the hemodialysis center of the Far Eastern Memorial Hospital had been conducted from February 2007 to July 2011. At the start of this period, a total of 374 patients were receiving maintenance hemodialysis, but excluded from this study were patients with (i) active infection, (ii) recent hospitalization within three months, (iii) psychotic illness or other communication problems, (iv) primary skin disorders, (v) cholestatic liver disease or acute hepatitis, or (vi) active malignancy. Finally, 321 patients (age: 60±12 years; 162 females and 159 males) were thus recruited in February 2007 [9], [13]. By the end of follow-up, there were 210 dropouts, including 96 withdrawals, 66 deaths, 3 renal transplantations, and 45 patients transferred to other hemodialysis centers. A total of 111 patients remained until the follow-up in July 2011 and completed the study. The study participants received 3.5–5.0 hours of hemodialysis three times a week using bicarbonate dialysate and reverse osmosis purified water, with the target dose of Kt/V (amount of dialysis delivered: K = clearance of urea, t = time on dialysis, V = estimated total body water) ≥1.4 to ensure the adequacy of solute clearance [17]. In 73% of participants, a high-flux polysulfone membrane was used as the dialyzer, while the remaining 27% used a low-flux synthetic membrane dialyzer.

### Ethics

This study was approved by the Institutional Review Board of Far Eastern Memorial Hospital in advance and a written informed consent was obtained from each participant.

### Pruritus Assessment

The severity of pruritus measured by the visual analogue scale (VAS) from 0 to 10 (0 = no pruritus, 10 = intolerable pruritus) was reported from each participant at baseline and follow-up. The evaluation of the baseline VAS score for each participant was completed in February 2007. In July 2011, the participants were re-evaluated using the VAS score to assess the severity of pruritus.

### Patient Characteristics and Laboratory Parameters

Patient demographic and clinical characteristics, including gender, age, presence of hypertension or diabetes, underlying renal disease, concurrent medications, as well as the regimens and vintage of hemodialysis, were recorded. Venous blood was sampled in the morning, after an overnight fast exceeding 8 hours before the patient’s mid-week dialysis. All laboratory tests were performed by the hospital’s central laboratory. Biochemistry data were determined using a Hitachi 747 auto-analyzer. The Kt/V and normalized protein catabolic rate (nPCR) were calculated using a single-compartment model [17], [18].The high sensitivity C-reactive protein (hs-CRP) levels were assayed by an image autoanalyzer using the nephelometric method (Beckman Coulter, Inc., CA, U.S.A.). The clinical and laboratory parameters were recorded for each participant at baseline in February 2007 and at follow-up in July 2011.

### Statistical Analysis

Statistical analysis was performed using the R 2.14.1 software (R Foundation for Statistical Computing, Vienna, Austria). A two-sided *P* value ≤0.05 was considered statistically significant. The distributional properties of continuous data were expressed as mean ± standard deviation or median with interquartile range, as appropriate. The categorical data were presented by frequency and percentage. As a descriptive analysis, univariate analyses were performed using a Student’s *t* test, Wilcoxon signed-rank test, Mann-Whitney *U* test, McNemar’s test, or Pearson Chi-square test, as appropriate.

Each participant’s clinical characteristics, laboratory parameters, and VAS score of pruritus intensity were recorded at baseline and follow-up. The intra-individual differences (called the *change score*) of these variables between baseline and follow-up were calculated, i.e., the change score of *X* = *X* at follow-up − *X* at baseline [19]. Causal analysis of individual change using the change score was performed [19], which allows causal interpretation of relationships between individual characteristics and patterns of uremic pruritus intensity [20]. In the multivariate analysis, a *change-score analysis* was conducted using the linear regression model to identify the predictors associated with the change score of VAS [20]. Given the available covariates including age, gender, comorbid diseases, and blood laboratory parameters, the regression analysis of change score of VAS proceeded in two steps: (1) only the baseline covariates, not the change scores of covariates, were considered in regression analysis; (2) next, both the baseline and change scores of covariates were considered in regression analysis.

Generalized additive models (GAMs) are able to detect nonlinear effects of continuous covariates during the stepwise variable selection procedure of regression analysis [21]. We used GAMs to discover the functional form of the potential dose-response relationship between baseline Kt/V and change score of VAS after adjusting for the effects of confounding factors. Computationally, we used the vgam function (with its default values of smoothing parameters) of the VGAM package [22], [23] and the gam function (with its default values of smoothing parameters) of the gam package to fit GAMs of VAS change score [24], [25].Then, the appropriate cut-off point for identifying the threshold effect was chosen by locating the value at which the smoothed curve crossed the horizontal line of *Y* axis = 0 in the GAM plot. The receiver operating characteristic (ROC) curve and the Youden index was used to verify the appropriateness of the chosen cut-off point of baseline Kt/V for predicting the aggravation of pruritus [26]. Additional linear regression analysis was also conducted to incorporate this threshold effect.

To ensure the quality of analysis results, basic model-fitting techniques for (1) variable selection, (2) goodness-of-fit (GOF) assessment, and (3) regression diagnostics were used in our regression analyses. Specifically, the stepwise variable selection procedure (with iterations between the forward and backward steps) was applied to obtain the candidate final regression model. All the relevant covariates, such as the covariates measured at baseline and the change scores of covariates, were put on the variable list to be selected, and the significance levels for entry (SLE) and for stay (SLS) were set to 0.15 or larger to be conservative. Then, the best final regression model was identified manually by reducing the significance levels to 0.05. The coefficient of determination, *R* ^2^, was computed to assess the GOF of the fitted multiple linear regression model. Technically, the *R* ^2^ statistic (0≤*R* ^2^≤1) indicates how much of the response variability is explained by the covariates included in the fitted multiple linear regression model, and it equals the square of the Pearson correlation between the observed and predicted response values. Finally, the statistical tools for regression diagnostics such as residual analysis, detection of influential cases, and check for multicollinearity were used to discover model or data problems.

## Results

### Patient Characteristics

**Figure 1:**
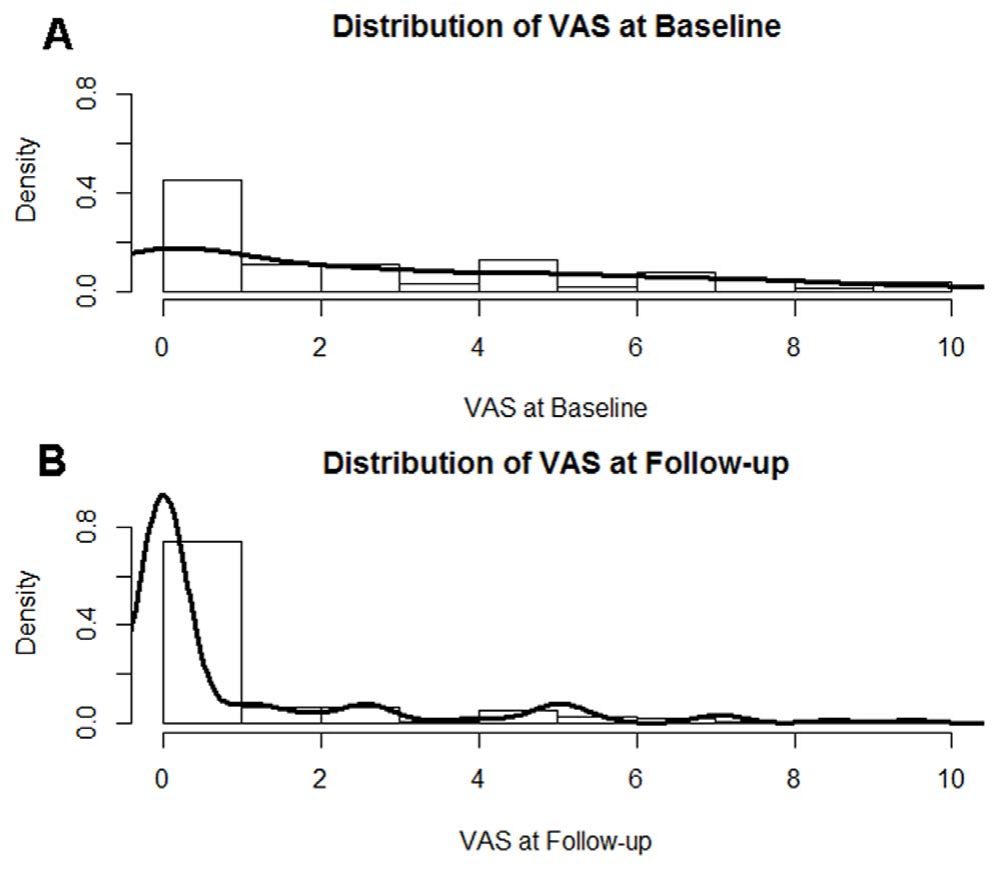
Histogram and density plot of visual analogue scale (VAS) scores. (A) Frequency distribution of pruritus VAS scores at baseline in the study participants. (B) Frequency distribution of pruritus VAS scores at follow-up in the study participants. The density of vertical axis represents the percentage of study participants.

**Figure 2:**
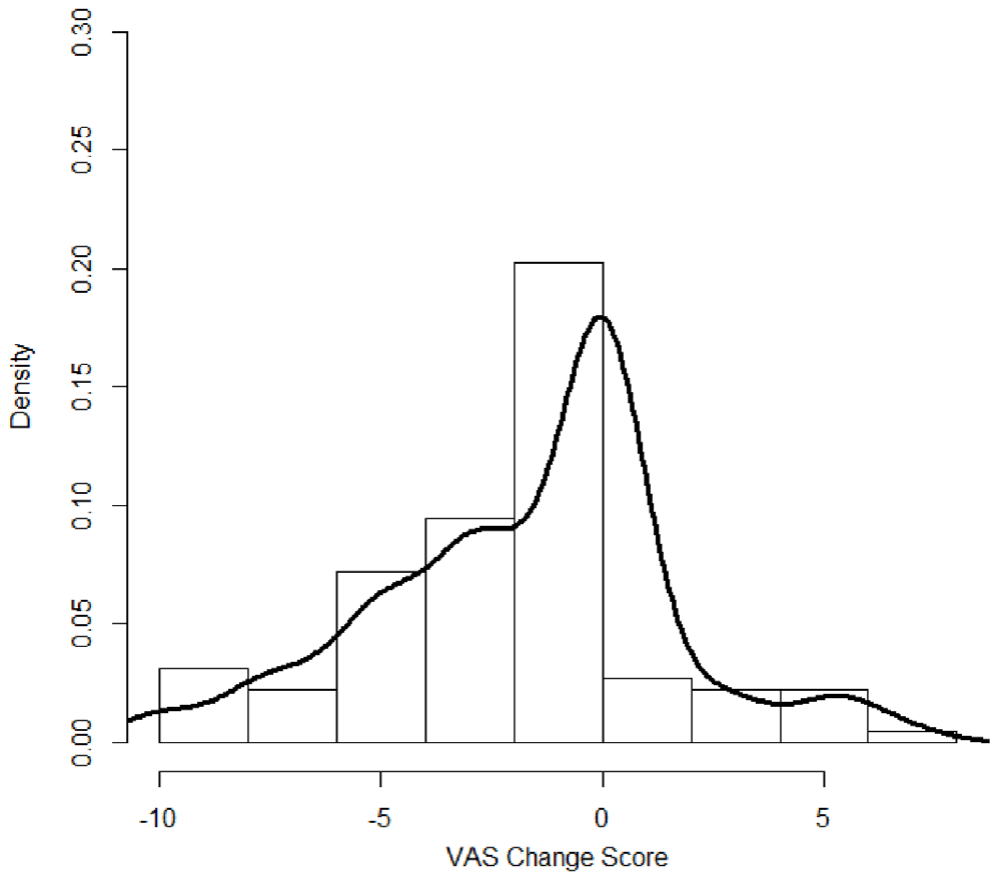
Histogram and density plot of visual analogue scale (VAS) change scores. Frequency distribution of pruritus VAS change scores in the study participants. The density of vertical axis represents the percentage of study participants. The VAS change score = VAS score at follow-up − VAS score at baseline.

A total of 111 patients completed this study, and their demographic and clinical characteristics are summarized in Table 1. The mean age of the study participants was 60.6±11.7 years, with 53% being female and 29% diabetic. The distribution of VAS scores at baseline and follow-up is shown in Figure 1. Table 2 shows the laboratory and clinical data of the study participants collected at baseline and at the end of the follow-up. Compared with the laboratory parameters measured at baseline, the blood levels of hematocrit, calcium, and alkaline phosphatase were higher, while the blood levels of leukocyte count and albumin were lower at follow-up (Table 2). Compared with the pruritus intensity at baseline, 17 patients (15.3%) reported aggravated pruritus, 37 patients (33.3%) reported unchanged pruritus, and 57 patients (51.4%) reported improved pruritus at follow-up. The distribution of VAS change score between baseline and follow-up is shown in Figure 2. The baseline VAS scores were not significantly different (*P* = 0.31) between the participants who completed the study (2.7±3.0) and those who dropped out (3.1±3.1).

**Table 1:** Demographic and clinical characteristics of participants at baseline.

Baseline characteristics	Statistics
Patient number (*n*)	111
Gender (female: male)	59 (53.2):52 (46.8)
Age (years)	60.6±11.7
Dialysis Vintage (years)	8.5±4.0
Use of high-flux dialyzer	81 (73.0)
Etiology of end stage renal disease (n)	
Diabetes mellitus	32 (28.8)
Glomerulonephritis	47 (42.3)
Hypertension	14 (12.6)
Obstructive uropathy	2 (1.8)
Tuberculosis	1 (0.9)
Others	15 (13.5)
Treatment for pruritus	
Systemic treatment	24 (21.6)
Topical medication	28 (25.2)
Body cream	25 (22.5)

NOTE. Data are expressed as mean ± S.D. or number (percentage).

**Table 2:** Laboratory and clinical characteristics of participants at baseline and follow-up.

Variable	Baseline	Follow-up	*P* Value
Participant number (n)	111	111	
VAS score of pruritusintensity	2.0 (5.0)	0.0 (1.2)	<0.001*
White blood cells (K/µL)	6.85±1.78	6.40±1.83	0.02*
Hematocrit (%)	33.0±4.3	34.5±4.0	<0.01*
Creatinine (mg/dL)	11.0±2.1	11.0±2.1	0.90
Uric acid (mg/dL)	7.6 (2.3)	7.5 (2.1)	0.85
Kt/V	1.59±0.23	1.57±0.19	0.33
Albumin (g/dL)	4.2 (0.4)	4.1 (0.3)	<0.01*
Fasting glucose (mg/dL)	97.0 (64)	102.0 (62)	0.33
Total cholesterol (mg/dL)	176.8±44.0	183.0±45.4	0.053
Triglyceride (mg/dL)	147.0 (158.0)	122.0 (121.0)	0.11
Aspartate transaminase (U/L)	17.0 (10.0)	16.0 (10.0)	1.00
Alanine transaminase (U/L)	14.0 (10.0)	12.0 (9.0)	0.07
Alkaline phosphatase (U/L)	74.0 (38.0)	81.0 (54.0)	0.02*
Total bilirubin (mg/dL)	0.3 (0.2)	0.3 (0.2)	0.34
Ferritin (ng/mL)	511.7 (267.0)	543.1 (250.7)	0.63
Calcium, albumin adjusted(mg/dL)	9.1 (0.8)	9.4 (1.0)	<0.01*
Phosphorus (mg/dL)	5.0 (1.8)	5.1 (1.3)	0.57
Ca×P** (mg/dL×mg/dL)	45.6 (17.1)	48.6 (13.8)	0.57
Intact parathyroid hormone(pg/mL)	249.0 (320.0)	334.0 (456.0)	0.29
C-reactive protein (mg/L)	0.25 (0.62)	0.28 (0.50)	0.21
Diabetes Mellitus	32 (28.8)	32 (28.8)	1.00
Hepatitis B	20 (18.0)	20 (18.0)	1.00
Hepatitis C	10 (9.0)	12 (10.8)	0.16

NOTE. Data are expressed as mean ± S.D for normally distributed continuous variables; as median (interquartile range) for non-normally distributed continuous variables; and as number (percentage) for categorical variables.

Abbreviations: VAS, visual analog scale.

* *P*<0.05 for the statistical testing between baseline and follow-up.

** Ca×P = Product of albumin-adjusted serum calcium (Ca) and serum phosphorus (P).

Table 3 shows the laboratory and clinical data of the study participants with improved and unimproved pruritus severity during the follow-up period. The baseline VAS score was higher in patients with improved pruritus (4.9±2.6) than those with unimproved pruritus (0.5±1.2). Patients with improved pruritus had lower blood levels of phosphorus, ferritin, as well as product of calcium and phosphorus, than those with unimproved pruritus at follow-up.

**Table 3:** Laboratory and clinical characteristics of participants with improved and unimproved pruritus intensity, at baseline and at follow-up.

	Baseline (2007)			Follow-up (2011)		
	Improvedpruritus	Unimprovedpruritus	*P* value	Improvedpruritus	Unimprovedpruritus	*P* value
Participant number (n)	57	54		57	54	
Gender (female : male)	27 (47):30 (53)	32 (59) : 22 (41)	0.21	27 (47):30 (53)	32 (59):22 (41)	0.21
Age (year)	56.0±12.6	57.2±10.6	0.60	60.0±12.6	61.2±10.6	0.60
VAS score of pruritus intensity	5.0 (4.0)	0.0 (0.0)	<0.01*	0.0 (0.3)	0.0 (2.6)	0.12
White blood cells (K/µL)	6.79±1.71	6.91±1.86	0.73	6.51±1.97	6.27±1.67	0.50
Hematocrit (%)	33.5±4.4	32.5±4.1	0.24	34.1±4.2	34.9±3.7	0.32
Creatinine (mg/dL)	10.9±2.1	11.1±2.1	0.58	10.9±2.2	11.2±1.9	0.49
Uric acid (mg/dL)	7.4 (2.3)	7.6 (2.2)	0.86	7.4 (2.2)	7.8 (2.0)	0.27
Kt/V	1.62±0.23	1.56±0.24	0.18	1.71±0.83	1.54±0.17	0.13
Albumin (g/dL)	4.2 (0.3)	4.2 (0.4)	0.43	4.1 (0.4)	4.1 (0.4)	0.31
Fasting glucose (mg/dL)	89.0 (49.0)	117.0 (72.8)	0.15	102.0 (59.5)	102.5 (66.0)	0.85
Total cholesterol (mg/dL)	179.7±43.2	178.8±45.1	0.49	175.6±42.7	190.8±47.1	0.08
Triglyceride (mg/dL)	139.0 (128.5)	152.5 (190.3)	0.54	107.0 (91.0)	129.5 (188.0)	0.23
Aspartate transaminase (U/L)	18.0 (11.0)	16.0 (11.0)	0.56	16.0 (8.0)	17.0 (12.0)	0.19
Alanine transaminase (U/L)	15.0 (10.0)	13.5 (10.0)	0.42	12.0 (8.0)	12.0 (10.0)	0.92
Alkaline phosphatase (U/L)	75.0 (40.5)	73.5 (37.0)	0.73	81.0 (54.5)	81.5 (54.5)	0.96
Total bilirubin (mg/dL)	0.3 (0.2)	0.3 (0.2)	0.59	0.3 (0.2)	0.3 (0.2)	0.63
Ferritin (ng/mL)	535.0 (392.2)	495.2 (229.8)	0.58	507.8 (274.1)	560.1 (287.6)	0.04*
Calcium, albumin adjusted (mg/dL)	9.0 (0.8)	9.2 (0.8)	0.24	9.3 (0.8)	9.5 (1.1)	0.26
Phosphorus (mg/dL)	4.7 (1.7)	5.1 (1.6)	0.13	4.8 (1.5)	5.3 (1.1)	0.04*
Ca×P** (mg/dL×mg/dL)	45.3 (18.3)	47.2 (15.7)	0.09	45.5 (12.3)	50.6 (11.8)	0.02*
Intact parathyroid hormone (pg/mL)	248.0 (290.5)	258.5 (393.0)	0.67	336.0 (440.5)	323.0 (493.1)	0.81
C-reactive protein (mg/L)	0.36 (0.88)	0.18 (0.39)	0.06	0.41 (0.61)	0.21 (0.28)	0.14

NOTE. Data are expressed as mean ± S.D for normally distributed continuous variables; as median (interquartile range) for non-normally distributed continuous variables; and as number (percentage) for categorical variables.

Abbreviations: VAS, visual analog scale.

* *P*<0.05 for the statistical testing between participants with improved pruritus and those with unimproved pruritus.

** Ca×P = Product of albumin-adjusted serum calcium (Ca) and serum phosphorus (P).

### Factors Associated with Aggravation of Pruritus Intensity Over Time

As listed in Tables 4, the regression analysis proceeded in two steps to identify the predictors associated with the aggravation of pruritus intensity over time. Regression model 1 considered only the covariates measured at baseline, including gender, age, VAS score of pruritus intensity, laboratory parameters, Kt/V, as well as regimens and vintage of hemodialysis (Table 4). We found that female gender, use of low-flux dialyzer, lower Kt/V at baseline, lower blood levels of uric acid at baseline, lower baseline VAS score, and higher blood levels of creatinine and preprandial glucose at baseline were significantly associated with the aggravation of pruritus intensity. There was also a borderline positive association between higher blood level of total bilirubin at baseline and worsened pruritus (Table 4, model 1). This linear regression model had a good fit to the data (*R* ^2^ = 0.736).

**Table 4:** Linear regression analysis of predictors associated with the change score of pruritus intensity.

	Parameter	Standard	95% Confidence		
Covariate	Estimate	Error	Interval	*P* Value	
**Regression Model 1** 1 **:**					
Intercept	0.54	2.46	−4.28–−5.37	0.83	
Male gender	−1.43	0.47	−2.35–−0.51	0.003	
Kt/V	−3.02	0.92	−4.83–−1.21	0.002	
Use of high-flux dialyzer	−0.86	0.42	−1.68–−0.04	0.04	
VAS score of pruritus intensity at baseline	−0.85	0.06	−0.97–−0.73	<0.001	
Hematocrit (%)	0.06	0.05	−0.03–0.16	0.19	
Creatinine (mg/dL)	0.37	0.11	0.15–0.59	0.002	
Uric acid (mg/dL)	−0.32	0.14	−0.59–−0.05	0.02	
Fasting glucose (mg/dL)	0.010	0.003	0.003–0.016	0.004	
Total bilirubin (mg/dL)	2.19	1.31	−0.38–4.75	0.10	
Ca×P* (mg/dL×mg/dL)	0.01	0.02	−0.02–0.05	0.44	
**Regression Model 2** 2 **:**					
Intercept	0.63	1.50	−2.31–3.58	0.68	
Kt/V	−1.98	0.76	−3.48–−0.48	0.01	
VAS score of pruritus intensity at baseline	−0.85	0.06	−0.97–−0.73	<0.001	
AST (U/L)	0.07	0.02	0.03–0.12	0.002	
Ca×P* (mg/dL×mg/dL)	0.04	0.02	0.01–0.07	0.01	
Change score of uric acid (mg/dL)	0.27	0.12	0.05–0.50	0.02	
Change score of fasting glucose (mg/dL)	−0.008	0.002	−0.012–−0.004	<0.001	
Change score of AST (U/L)	0.13	0.03	0.07–0.19	<0.001	

NOTE. Each participant’s change score of pruritus intensity = follow-up VAS score of pruritus intensity – baseline VAS score of pruritus intensity. Similarly, each participant’s change score of a covariate = follow-up data of a covariate – baseline data of a covariate.

Abbreviations: AST, aspartate transaminase; VAS, visual analog scale.

* Ca×P = Product of albumin-adjusted serum calcium (Ca) and serum phosphorus (P).

1 Model considered only the covariates measured at baseline (gender, Kt/V, use of high-flux dialyzer, pruritus intensity, hematocrit, creatinine, uric acid, fasting glucose, total bilirubin, Ca×P). *R* ^2^ = 0.736.

2 Model considered both the covariates measured at baseline (Kt/V, pruritus intensity, AST, Ca×P) and the change scores of the covariates (uric acid, fasting glucose, AST). *R* ^2^ = 0.752.

Regression model 2 considered both the covariates measured at baseline and the change scores of covariates (Table 4). In addition to higher blood level of aspartate aminotransferase (AST) at baseline, lower Kt/V at baseline, and higher value in the product of calcium and phosphate at baseline, we found that higher change scores of uric acid and AST and a lower change score of fasting glucose were significantly associated with the aggravation of pruritus (Table 4, model 2). Compared to model 1, this linear regression model had a better fit to the data (*R* ^2^ = 0.752).

### The Threshold of Kt/V

**Figure 3:**
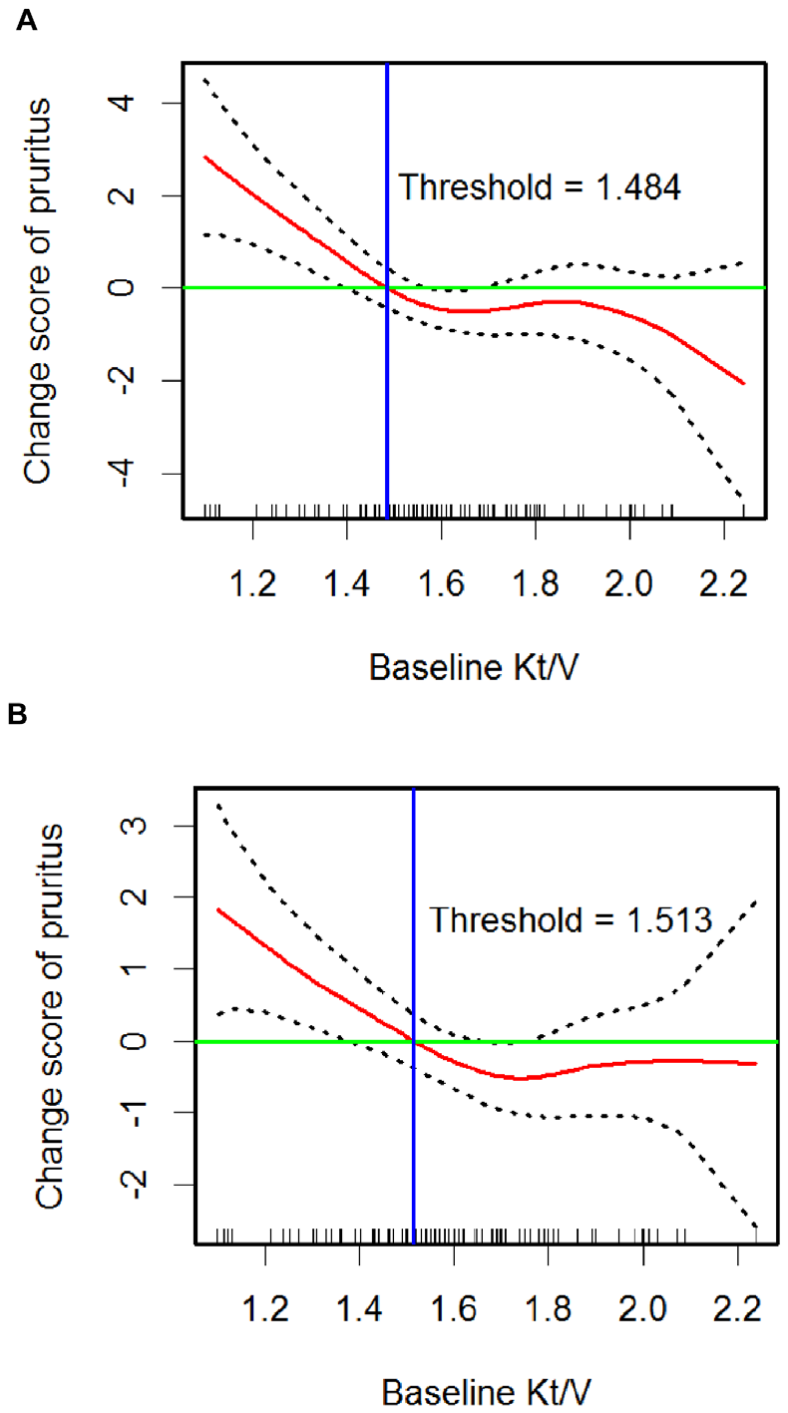
Identifying the appropriate threshold of baseline Kt/V by the generalized additive models (GAM) plot. (A) The GAM plot adjusted for the important covariates at baseline only (gender, Kt/V, use of high-flux dialyzer, pruritus intensity, hematocrit, creatinine, uric acid, fasting glucose, total bilirubin, and Ca×P).(B) The GAM plot adjusted for the important covariates at baseline (Kt/V, pruritus intensity, AST, and Ca×P) and the change scores of the covariates (uric acid, fasting glucose, and AST). The solid red lines show nonlinearity of multivariable-adjusted relation between baseline Kt/V and change score of pruritus (with 95% confidence intervals shown in black dotted lines). Pruritus intensity was assessed by visual analog scale scores. The little vertical bars (i.e., rugs) on the horizontal axis of the GAM plots display the distribution of individual observations. Both GAM plots identified the value around 1.5 to be the appropriate threshold of baseline Kt/V for uremic pruritus, which indicated start of the aggravation of pruritus intensity began at Kt/V <1.5.

The GAM plots adjusted for baseline covariates with and without the change scores of covariates both revealed that baseline Kt/V was negatively and approximately linearly associated with the change score of VAS (Figure 3). In the absence of evidence to determine a threshold value in the continuum of Kt/V for uremic pruritus, we sought an appropriate cut-off value of the baseline Kt/V associated with the aggravation of pruritus. In both GAM plots, the functional dose of Kt/V (in red) intersected the zero VAS score changes (in green) at a value of around 1.5. These intersections indicate that a baseline Kt/V <1.5 is associated with the aggravation of pruritus intensity.

To further verify the appropriate threshold of Kt/V, we have examined various cut-points of Kt/V on the dichotomous outcomes of whether pruritus improved or not. The receiver operating characteristic (ROC) curve showed an area under the curve of 0.587 (95% confidence interval 0.480–0.695). By using the Youden index, we have identified 1.515 to be the optimal cut-point of Kt/V, with a sensitivity of 73.7% and specificity of 50%. This cut-point is nearly the same as we have identified from the GAM plots. Therefore 1.5 should be an appropriate threshold value of Kt/V for the aggravation of pruritus intensity.

### Categorizing Participants by Kt/V at the Threshold of 1.5

According to the threshold determined by the GAM plots and ROC curve, we further classified the participants into two categories: those with baseline Kt/V <1.5 and those with baseline Kt/V ≥1.5. We then re-evaluated the predictors associated with the aggravation of pruritus intensity in the regression model considered only the covariates measured at baseline (Table 5, model 3), and the model considered both the covariates measured at baseline and the change scores of covariates (Table 5, model 4). Models 3 and 4 (Table 5) identified the same set of significant covariates associated with worsened pruritus as those of models 1 and 2 (Table 4), respectively. Moreover, they had compatible GOF as indicated by *R* ^2^.

**Table 5:** Linear regression analysis of the predictors associated with the change score of pruritus intensity with dichotomized baseline Kt/V at its appropriate threshold value of 1.5.

	Parameter	Standard	95% Confidence			
Covariate	Estimate	Error	Interval		*P* Value	
**Regression Model 3** 1 **:**						
Intercept	−2.67	1.30	−5.23–−0.12	0.04		
Male gender	−1.17	0.44	−2.03–−0.30	0.01		
Kt/V <1.5	1.27	0.42	0.44–2.09	0.004		
Use of high-flux dialyzer	−0.85	0.42	−1.67–−0.02	0.047		
VAS score of pruritus intensity at baseline	−0.81	0.06	−0.93–−0.69	<0.001		
Creatinine (mg/dL)	0.42	0.11	0.19–−0.64	<0.001		
Uric acid (mg/dL)	−0.42	0.14	−0.69–−0.15	0.003		
Fasting glucose (mg/dL)	0.011	0.003	0.004–0.017	0.001		
Phosphorus (mg/dL)	0.26	0.15	−0.03–0.55	0.09		
**Regression Model 4** 2 **:**						
Intercept	−3.01	0.93	−4.83–−1.18	0.002		
Kt/V <1.5	1.02	0.37	0.29–1.74	0.01		
VAS score of pruritus intensity at baseline	−0.83	0.06	−0.95–−0.72	<0.001		
AST (U/L)	0.07	0.02	0.03–0.11	0.003		
Ca×P* (mg/dL×mg/dL)	0.04	0.02	0.01–0.07	0.01		
Change score of uric acid (mg/dL)	0.29	0.12	0.06–0.52	0.01		
Change score of fasting glucose (mg/dL)	−0.008	0.002	−0.012–−0.004	<0.001		
Change score of AST (U/L)	0.13	0.03	0.07–0.19	<0.001		

NOTE. Each participant’s change score of pruritus intensity = follow-up VAS score of pruritus intensity – baseline VAS score of pruritus intensity. Similarly, each participant’s change score of a covariate = follow-up data of a covariate – baseline data of a covariate. The appropriate threshold of Kt/V, determined by generalized additive models and receiver operating characteristic analysis, was 1.5. Accordingly, the participants were classified into two categories: those with Kt/V <1.5 and those with Kt/V ≥1.5.

Abbreviations: AST = Aspartate transaminase; VAS = Visual analog scale.

* Ca×P = Product of albumin-adjusted serum calcium (Ca) and serum phosphorus (P).

1 Model adjusted for covariates of baseline data only (gender, Kt/V, use of high-flux dialyzer, pruritus intensity, creatinine, uric acid, fasting glucose, phosphorus), *R* ^2^ = 0.725.

2 Model adjusted for the important covariates at baseline (Kt/V, pruritus intensity, AST, and Ca×P) and the change scores of the covariates (uric acid, fasting glucose, and AST), *R* ^2^ = 0.754.

## Discussion

This is the first prospective cohort study to investigate clinical parameters and metabolic profiles for uremic pruritus in hemodialysis patients using repeatedly measured data. In the study, we have demonstrated that the aggravation of pruritus was associated with lower Kt/V after adjusting for a variety of confounding factors. We’ve also shown that the patients with baseline Kt/V below 1.5 suffered from aggravation of pruritus intensity.

The relationship between uremic pruritus and the doses of hemodialysis remains controversial. Kt/V, the most commonly used indicator for dialysis adequacy, assesses the clearance of small molecules in uremic patients. Dyachenko and Hiroshige reported that achieving a higher Kt/V might reduce the degree of pruritus in hemodialysis patients [4], [27], while studies reported by Zucker or Duque did not support this beneficial effect [3], [28]. However, the conclusions by Zucker or Duque were limited due to no adjustment of other confounding factors.

According to current guidelines, the target dose of Kt/V for hemodialysis given three times weekly should be l.4 or above [17]. These guidelines are based on randomized clinical trials or large-scale observational studies mainly investigating the outcomes of all-cause mortality, but not quality of life or intensity of uremic pruritus [29]–[33]. Currently, there are no established guidelines or consensuses regarding the optimal levels of Kt/V to ameliorate pruritus in hemodialysis patients. The cut-off value of 1.5 for Kt/V suggested by our study for pruritus is slightly above the target levels of reducing mortality recommended in clinical practice guidelines, which not only implies that a Kt/V higher than the current standard may not further improve survival but may improve the quality of life, but also indicates that the clearance of pruritogenic substances could influence the intensity of pruritus [4], [34], [35]. In addition, we have found that the use of a high-flux dialyzer was associated with alleviation of pruritus intensity. Compared with low-flux dialyzers, a high-flux dialyzer more efficiently removes middle molecules ranging in size from 1000 to 15,000 D and has been shown to be associated with the improvement of plasma lipolytic activities [36], as well as lower rates of amyloidosis and mortality [37], [38]. Our study results are consistent with previous studies in demonstrating that uremic patients with pruritus have higher blood levels of urea nitrogen and β_2_-microglobulin than do patients without pruritus [4], [8]. Nonetheless, further studies are warranted to identify pruritogenic substances and potentially novel targets in order to help relieve uremic pruritus.

This study has several limitations. First, we only analyzed the participants who completed the follow-up of 5 years in the cohort study, which was limited by the number of our study participants and possibly influenced by informative censoring. Second, data-dependent model building and GAM plot construction intrinsically introduce several biases, including selection bias. The cut-off value of baseline Kt/V from our study has only been tested in the data set from which it was derived. External validation in other cohorts, as well as randomized clinical trials, are necessary to definitively determine whether increasing dialysis dose relieves uremic pruritus and to accurately identify the optimal dialysis dose and regimen. Third, most of our study participants presented milder pruritus, with the characteristics of pruritus intensity similar to other studies in recent years [16], [28], [39]. Further studies may be necessary to explore the factors specifically associated with more severe form of uremic pruritus. Fourth, this study was limited to the patients receiving chronic hemodialysis. Whether the findings are also applicable to the patients with chronic kidney disease or chronic peritoneal dialysis requires the verification of additional studies.

Despite these limitations, the present study has several strengths. This is the first prospective cohort study designed to explore the aggravating and relieving factors associated with uremic pruritus. The longitudinal study design and repeatedly measured covariates offer several advantages in terms of providing information about the temporal aspect of each participant, increased statistical power, and estimates of a greater range of conditional probabilities. As pruritus is a subjective feeling and generally considered difficult to evaluate, it is valuable to perform a longitudinal study to exclude time-invariant unobserved individual differences. Furthermore, information of a wide variety of potential confounding variables was collected repeatedly during follow-up, which allowed for more sufficient control of known potential confounders in our analysis.

In conclusion, our study demonstrates that dialysis adequacy assessed by Kt/V is an independent predictor of pruritus intensity in patients with maintenance hemodialysis. Furthermore, hemodialysis with the target of Kt/V ≥1.5, as well as the use of high-flux dialyzer, may play a role in reducing the severity of uremic pruritus.
