# Correction: Uremic Pruritus, Dialysis Adequacy, and Metabolic Profiles in Hemodialysis Patients: A Prospective 5-Year Cohort Study

**DOI:** 10.1371/annotation/43d10c8d-5a10-41c4-ad44-605dfee7f630

**Published:** 2013-10-01

**Authors:** Mei-Ju Ko, Hon-Yen Wu, Hung-Yuan Chen, Yen-Ling Chiu, Shih-Ping Hsu, Mei-Fen Pai, Ju-Yeh Yang, Chun-Fu Lai, Hui-Min Lu, Shu-Chen Huang, Shao-Yu Yang, Su-Ying Wen, Hsien-Ching Chiu, Fu-Chang Hu, Yu-Sen Peng, Shiou-Hwa Jee

The names of the seventh and 12th authors were spelled incorrectly. The correct names of the seventh and 12th authors are Ju-Yeh Yang and Su-Ying Wen, respectively.

In addition, multiple funding organizations were incorrectly given, and multiple funding organizations and grants were incorrectly omitted from the Funding Statement. The Funding Statement should read: "This study was supported by research grants to Dr. Mei-Ju Ko, from the National Science Council of Taiwan (NSC 102-2314-B-532-002), Department of Health, Taipei City Government (Taipei, Taiwan), and Taipei City Hospital, Ren-Ai branch (Taipei, Taiwan). The funders had no role in study design, data collection and analysis, decision to publish, or preparation of the manuscript." 

